# Lost in Translation: Challenges in the Diagnosis and Treatment of Early-Onset Schizophrenia

**DOI:** 10.7759/cureus.39488

**Published:** 2023-05-25

**Authors:** Nihit Gupta, Mayank Gupta, Michael Esang

**Affiliations:** 1 Psychiatry, Dayton Children's Hospital, Dayton, USA; 2 Psychiatry and Behavioral Sciences, Southwood Psychiatric Hospital, Pittsburgh, USA; 3 Psychiatry and Behavioral Sciences, Clarion Psychiatric Center, Clarion, USA

**Keywords:** prodromal psychosis, child and adolescent psychiatry, schizophrenia and other psychotic disorders, childhood-onset schizophrenia, very early onset schizophrenia (veos)

## Abstract

Early-onset schizophrenia (EOS) is a heterogeneous condition that has a serious, insidious clinical course and poor long-term mental health outcomes. The clinical presentations are highly complex due to the overlapping symptomatology with other illnesses, which contributes to a delay in the diagnosis. The objective of the review is to study if an earlier age of onset (AAO) of EOS has poor clinical outcomes, the diagnostic challenges of EOS, and effective treatment strategies. The review provides a comprehensive literature search of 5966 articles and summarizes 126 selected for empirical evidence to methodically consider challenges in diagnosing and treating EOS for practicing clinicians. The risk factors of EOS are unique but have been shared with many other neuropsychiatric illnesses. Most of the risk factors, including genetics and obstetric complications, are nonmodifiable. The role of early diagnosis in reducing the duration of untreated psychosis (DUP) remains critical to reducing overall morbidity. Many specific issues contribute to the risk and clinical outcomes. Therefore, issues around diagnostic ambiguity, treatment resistance, nonadherence, and rehospitalizations further extend the DUP. There is hesitancy to initiate clozapine early, even though the empirical evidence strongly supports its use. There is a growing body of research that suggests the use of long-acting injectables to address nonadherence, and these measures are largely underutilized in acute settings. The clinical presentations of EOS are complex. In addition to the presence of specific risk factors, patients with an early onset of illness are also at a higher risk for treatment resistance. While there is a need to develop tools for early diagnosis, established evidence-based measures to address nonadherence, psychoeducation, and resistance must be incorporated into the treatment planning.

## Introduction and background

First-episode psychosis (FEP) is also used interchangeably with early-onset schizophrenia (EOS), although only a fraction of FEP eventually evolves into schizophrenia. Although EOS [1] and childhood-onset schizophrenia (COS) are not separate terms in the DSM-5 [2], they are extensively investigated independently. COS is an extremely rare condition [3], with a prevalence as low as 0.05% below the age of 13 [4], and the EOS’s onset is typically in mid-adolescence, accounting for the majority of the cases [5,6].

In the last two decades, instruments have been developed to identify these At-Risk Mental States (ARMS) for predicting a later transition to a psychotic disorder. However, given the heterogeneous nature of clinical presentations, many of these experiences are often transient. A recent meta-analysis has recommended against the use of the Comprehensive Assessment of At-Risk Mental States (CAARMS) and the Structured Interview for Psychosis Risk Syndromes (SIPS) to identify these high-risk individuals [7]. Since psychotic-like experiences occur in 6% to 8% of children and adolescents and in up to 28% of adults, the 1990s term "clinical high risk for psychosis" [CHR-P] or the similar DSM-5 diagnostic construct "attenuated psychosis syndrome" [APS] is often used to describe epidemiological data of at-risk individuals in the scientific literature. Although there are instruments like the Schizophrenia Proneness Instrument, Child and Youth Version (SPI-CY), and the Prodromal Questionnaire-Brief Child Version (PQ-BC), which are useful measures of early risk for psychotic disorders, their use in clinical practice is rare [8,9].

There are many individuals with autism spectrum disorders (ASD) who present with psychotic symptoms earlier; therefore, ASD must not be considered a mutually exclusive diagnosis in CHR individuals [10]. The presence of non-affective psychosis (like schizophrenia) in ASD individuals is poorly understood [11], and emerging data from genome-wide association studies (GWAS) with higher polygenic risk (PRS) for scores for both schizophrenia and ASD remains a focus of future research. It is widely debated that EOS phenomenology is indistinguishable from other illnesses [12,13]; has a more severe debilitating course [14,15], and is often refractory to treatment [16,17]. About 25% of EOS patients were treatment-resistant even at the time of onset of the illness [18,19].

In clinical settings, the presentations are complex with many symptom proxies and high comorbidity, leading to diagnostic and nosologic difficulties and thereby affecting the overall clinical outcomes. Therefore, we reviewed empirical literature following a search using a pointed and narrow criterion for this narrative review. There are three key aims of this review: first, to understand if the earlier age of onset (AAO) of EOS has poor clinical outcomes. And second, what are the specific risk factors, and lastly, what are the evidence-based strategies for early-onset treatment-resistant schizophrenia (TRS)?

Method

**Figure 1:**
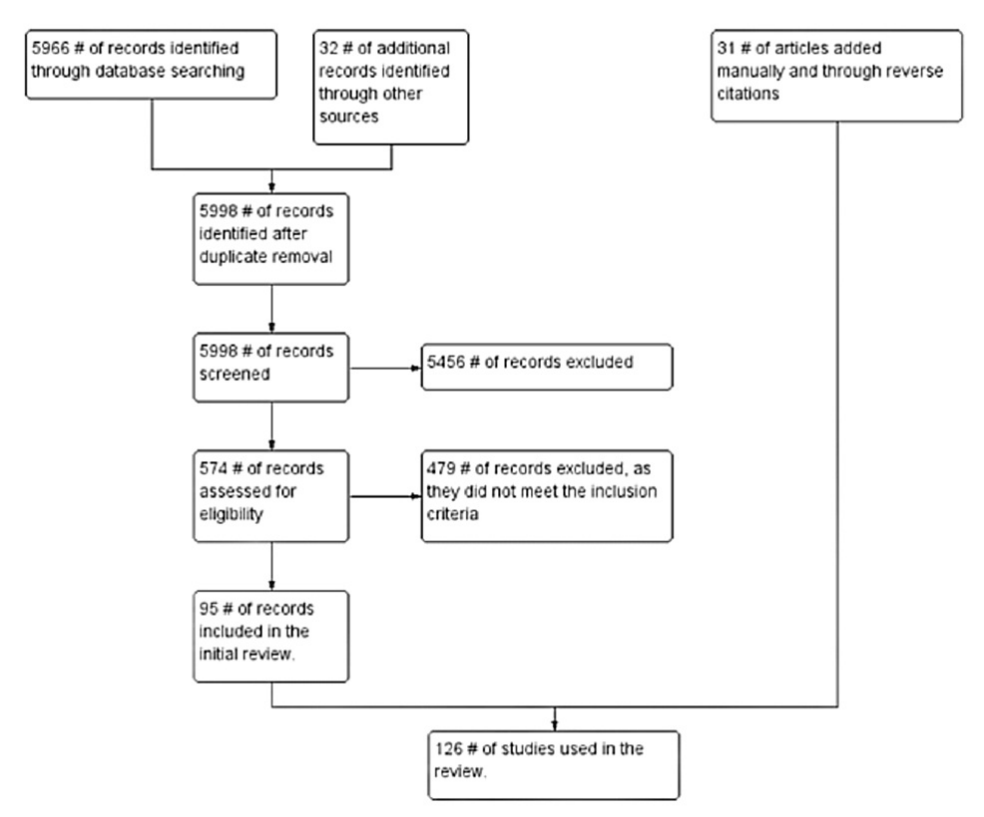
Search strategy for the review

Our review of the literature involved searching three electronic databases (PubMed, Google Scholar, and Cochrane). The analysis was conducted using the controlled vocabulary and keywords: "early-onset schizophrenia," "prodromal psychosis," "childhood-onset psychosis," "schizophreniform disorder," "first-episode psychosis," and "duration of untreated psychosis." Studies focusing exclusively on other primary psychiatric disorders were excluded. Studies with all age groups, co-occurring mental health conditions, and other substance use were included in the review. The search was conducted by all authors, and if there were disagreements regarding the inclusion or exclusion of papers, a consensus was reached through discussion amongst all the authors. The authors included studies that they thought would be beneficial in educating practitioners about early-onset psychosis. This review only included studies on human subjects published in English-language journals or those with official English translations. Studies that were included in this manuscript were not restricted by the date of publication. The PRISMA guidelines were followed and adhered to (Figure 1).

## Review

We have subdivided this section based on the review of the literature to focus on the key domains.

Risk factors specific to EOS

The role of genetics [20] is critical in altering neurodevelopmental trajectories [1] and has been attributed to worse clinical outcomes [21]. The concordance rate of COS in monozygotic twins was 88.2% compared to 22.3% in dizygotic twins, with an overall high genetic heritability of 84.5% [22]. When 130 COS probands were compared to 103 siblings, they had a higher PRS for scores for both schizophrenia and ASD [20]. Elevated rates of large copy number variants (CNVs) have also been found in COS, including in CNVs associated with schizophrenia and other neurodevelopmental disorders [23]. The rates of large and rare CNVs appear to be higher in COS patients in comparison to both controls and adults with adult-onset schizophrenia (AOS) [23,24]. About 11.9% of COS probands were estimated to have a neurodevelopmental disease-associated CNV, compared to 1.5% of their healthy siblings and 1.4-4.9% of AOS [25]. A high number of COS probands have CNVs at the 22q11.2 locus, which is known to increase the risk for multiple psychiatric and neurodevelopmental disorders, including schizophrenia, ASD, ID (intellectual disability), and attention deficit hyperactivity disorder (ADHD) [26].

Premorbid factors such as hypoxemia related to obstetric complications [27,28], and IQ < 85 [29] are associated with the early onset [30]. In an interesting study of placental genomics, the schizophrenia PRS was five times higher with early-life complications (ELCs) [31].

GWAS have provided insight into the genetic contribution and multiple statistically independent signals of neuropsychiatric disorders. In a groundbreaking work, 25 disorders of the brain from GWA studies of 265,218 patients and 784,643 controls were assessed for the relationship of 17 phenotypes in 1,191,588 individuals [32]. The study found psychiatric disorders share a common variant (CV) risk, whereas neurological disorders appear more distinct from each other than psychiatric disorders.

In-utero exposure to starvation in women has a high odds of risk [33,34]. Folic acid supplantation is associated with altered cortical development and a reduced risk of autism [35-37]. Likewise, there is an association between gestational use of folic acid and a reduction in the risk of psychosis in youth [38]. ASD and schizophrenia are two distinct behavioral outcomes of aberrant neurodevelopment, and their differentiation is frequently easy, clinically useful, and categorical nosology. However, the boundaries are not always clear, and several counts of evidence from phenomenology, epidemiology, genetics, and neuroscience point toward a close relationship between the two disorders [39]. Lastly, another study links patients with weekly to daily cannabis use before illness onset with the highest PRS for schizophrenia (p = 0.02, Cohen's d = 0.33) [40]. The findings are highlighted in Table 1.

**Table 1:** Risk factors specific to early-onset schizophrenia CNV: copy number variants, COS: childhood-onset schizophrenia, EOS: early-onset schizophrenia

Author and year	Key findings
Ahn et al. [20], Harvey et al. [21], Kallman et al. [22]	Genetic predisposition to EOS leads to worse clinical outcomes with an overall heritability of 84.5%.
Ahn et al. [23], Nicolas et al. [24], Sagar et al. [25], Vorstman et al. [26]	COS is associated with elevated rates of large and rare CNVs.
Byrne et al. [27], Rosso et al. [28], Díaz-Caneja et al. [29], Baeza et al. [30], Ursini et al. [31]	Premorbid factors like birth hypoxia and IQ < 85 are associated with increased risk for early onset.
St Clair et al. [33], Susser et al. [34], Levine et al. [35], Eryilmaz et al. [38]	In utero exposure to starvation in women has a higher odds of risk while gestational folic acid use is associated with a reduction in the risk of psychosis among youths.
Aas et al. [40]	The highest risk is with individuals who use cannabis weekly or daily.

Clinical profiles of EOS and duration of untreated psychosis

EOS is a highly heterogeneous disorder with a younger AAO, and the presence of bizarre positive symptoms is suggestive of a severe course [41]. In many demographics, the clinical profiles of EOS and the scope of the problem are not yet determined [42]. The prevalence increases rapidly after the age of 14 years, particularly in males, and accounts for about 25% of all psychiatric admissions in young people between 10 and 18 years of age. Besides the poorer prognosis of EOS, about 30% require long-term mental health services. The worst outcomes are associated with poorer premorbid functioning, an insidious onset, lower intellectual function, and the presence of negative symptoms [43]. Another study reports that longer follow-up periods, male sex, and being diagnosed before 1970 contribute to the poor course of the EOS [1]. There is a strong correlation between younger AAOs and more hospitalizations, more negative symptoms, a higher risk of relapses, poorer social/occupational functioning, and poorer global outcomes [44]. The risk of suicide for people with schizophrenia is higher in those with an earlier onset [45,46]. While the above prognostic indicators are not modifiable, one stands out as being amenable to intervention, that is the duration of untreated psychosis (DUP), which is associated with worse clinical outcomes [47-50], making early diagnosis and efficacious treatment crucial [51]. Key findings can be reviewed in Table 2.

**Table 2:** Clinical profiles of early-onset schizophrenia and duration of untreated psychosis EOS: early-onset schizophrenia

Author and year	Key findings
Giannitelli et al. [41]	Younger age of onset and bizarre positive symptoms suggests a severe course of illness
Abidi et al. [43]	Worse outcomes associated with poor premorbid functioning, insidious onset, lower intellectual functioning, and presence of negative symptoms
Olfson et al. [45], Bornheimer [46]	Higher risk of suicide in EOS
Vyas et al. [47], Fragus et al. [48]. Stentebjerg-Olesen et al. [49], Molina-García et al. [50], Coulon et al. [51]	Duration of untreated psychosis is associated with worse clinical outcomes

Advanced neuroimaging and diagnostics

The genes that code for schizophrenia also code for the thickness of the cerebral cortex [52]. Cortical thinning is a developmental process manifested by pruning and myelination. However, the thinning is accelerated in EOS, resulting in a smaller mean cortical thickness [53-54].

The gray matter (GM) deficits in the left prefrontal, insula, and bilateral temporal cortices and smaller deficits in the right prefrontal and inferior parietal cortices are more pronounced as compared to their age-controlled healthy counterparts [55-58]. Similar cortical deficits are found in siblings of patients with COS and are proposed as familial trait-makers for COS. In non-psychotic siblings, these gray matter deficits disappeared by age 20 [59]. The diagnostic "potential" of voxel-based morphometry (VBM), which estimates the distribution of gray matter tissue volume across several brain regions, has been studied with equivocal results [60,61]. Considering that VBM is evolving, it may offer a ray of hope, given the fact that clinical outcomes are highly dependent on treatment during the narrow window period. Table 3 summarizes the key features of neuroimaging and diagnostics in EOS.

**Table 3:** Advanced neuroimaging and diagnostics VBM: voxel-based morphometry, GM: gray matter, COS: childhood-onset schizophrenia

Author and Year	Key findings
Lee et al. [52], Ordóñez et al. [53], Greenstein et al. [54]	Accelerated cortical thinning leads to smaller mean cortical thickness
Vita et al. [55], Bartholomeusz et al. [56], Rapado-Castro et al. [57] Satterthwaite et al. [58]	The GM deficits in the left prefrontal, insula, and bilateral temporal cortices
Gogtay et al. [59]	Similar cortical deficits are found in siblings of patients with COS and are proposed as familial trait makers for COS
Palaniyappan et al. [60], Torres et al. [61]	VBM which estimates the distribution of gray matter tissue volume across several brain regions has been studied with equivocal results

Treatment strategies for EOS

Interestingly, the European Psychiatric Association recommends cognitive behavioral therapy (CBT) for the CHR-P group, followed by low-dose second-generation antipsychotics if psychological interventions are ineffective [62]. The United Kingdom’s National Institute for Health and Care Excellence (NICE) recommends: "Do not offer antipsychotic medication to people considered to be at increased risk of developing psychosis with the aim of decreasing the risk of or preventing psychosis [63]."

The core component of early intervention services (EIS) [64,65] is pharmacotherapy [66]. The randomized controlled trials (RCT) of first-generation antipsychotics (FGAs) have high rates of extrapyramidal symptoms (EPS) and sedation; therefore, FGAs should not be the first-line regimen [67,68]. The RCTs of Olanzapine, Risperidone, Aripiprazole, Quetiapine, Paliperidone, Asenapine, and Ziprasidone have all been shown to be effective [69-74]. Children and adolescents are at higher risk for extrapyramidal symptoms, prolactin elevations, sedation, weight gain, and metabolic effects [75]. Among SGA, ziprasidone’s cardiac profile was not favorable [76].

In a recent study, Pagsberg et al. conducted a post hoc analysis of data derived from the randomized, double-blinded, 12-week tolerability and efficacy of antipsychotics (TEA) trial at seven different sites across Denmark, comparing oral extended-release quetiapine and aripiprazole in adolescents <18 years of age with first-episode psychosis. The group concluded that early nonresponse at four weeks to aripiprazole or quetiapine extended-release in FEP is an indicator of future nonresponse and a rationale to switch [77]. The cutoff scores defining early nonresponse as <20% symptom reduction at week 2 or <30% symptom reduction at week 4 have the best predictive value for both nonresponse and nonremission and thereby provide a rationale for the switch to different antipsychotics.

The study also reported a similar predictive significance for the PANSS-6 (Positive and Negative Syndrome Scale-6) and the PANSS-30. Since PANSS-6 is easy to administer, it could be of immense value to the clinical team [77].

Treatment-resistant schizophrenia (TRS) accounts for almost 35% of the total cases [19,78] and with emerging evidence for a new debate about whether TRS is categorically different from treatment-responsive schizophrenia [79]. TRS may be a heritable condition due to variations in single nucleotides, which need further investigation [80]. Besides a higher risk of neutropenia and seizures as compared to adults, clozapine [81,82] is most effective in TRS. It is also suggested that a trial with olanzapine [83] be initiated before initiating clozapine. Clozapine showed superior efficacy in treating refractory EOS when compared with risperidone, olanzapine, and paliperidone [84]. Similarly, clozapine was superior in individuals who failed two trials of standard antipsychotics [85]. The clinical improvements were sustained during long-term follow-up. In about 90% of patients, sedation and hypersalivation were the most common complaints. Neutropenia was reported in 6-15% of cases but was usually transient, while agranulocytosis was rare (<0.1%). Seizures were also uncommon (<3%). Overall, the rate of discontinuation was low (3-6%) [84], and the long-term use of clozapine substantially decreased mortality [86]. In a Canadian retrospective study of 28 inpatients (64% female) receiving clozapine between January 2000 and December 2014, the mean age at clozapine initiation was 15.8 years. Twenty-three patients (82%) were taking clozapine at discharge, and of these, 22 patients (96%) experienced some improvement. There were no episodes of severe neutropenia, but a high rate of benign hematological adverse events was seen. The study concludes that most treatment-refractory children and adolescents requiring hospitalization improve with clozapine, and most were able to tolerate it despite high rates of largely manageable side effects [87].

Current guidelines [88] recommend the use of clozapine in EOS patients who have failed to respond to two adequate trials with different antipsychotics and provide detailed schedules of assessments to evaluate and assess potential ADRs both before initiation and throughout the treatment [89-92].

EOS presents with a unique set of challenges, first due to the ambiguity of symptoms overlapping with other disorders and diagnostic uncertainty during the early stages [93]. The APS, or prodromal stage, is indistinguishable from other illnesses [94], and there are no recommended treatments besides weak evidence for omega-3 fatty acids. Likewise, the DUP is also difficult to measure in clinical settings, although the last two decades of empirical research point toward its reduction as the single most effective strategy to improve outcomes [95,96]. Many risk factors, like familial genetics and a history of obstetric complications, are useful in the diagnostic formulations but are commonly shared with many other co-occurring psychiatric disorders and are nonmodifiable. In clinical settings, comorbidity with anxiety, affective disorders [97], ADHD [98], ASD [99,100], ID, and substance use are common. The presence of comorbidity affects both diagnostics and is also associated with unfavorable outcomes [101]. There is also a strong critique of the current practices, and clinicians' contribution to the delay in diagnosis cannot be ignored [102]. Since there is a critical period when early treatment may increase the odds of recovery, these findings are even more concerning [103]. These delays contribute to the DUP, and having support from families with a better level of premorbid functioning improves outcomes [104].

Another systematic review found clinicians use multiple antipsychotics or higher than recommended doses rather than the recommended clozapine [105,106]. This increased the DUP range from 1.1 to 9.7 years before the initiation of clozapine [107]. Some reports of delay in the initiation of clozapine may be a key determinant of nonresponse in TRS [108]. Non-adherence to antipsychotic medication in EOS and FEP is a severe issue with a multifactorial etiology [109,110], and cannabis use has been highly associated with medication nonadherence and the risk of relapse [111]. In a three-year prospective study, medication non-adherence was a key predictive factor for relapse [112]. Given these critical findings, a three-year naturalistic study suggested the use of long-acting injectable (LAI) antipsychotics as first-line treatment since they significantly reduce the risk of relapse and hospitalization in patients with poor prognostic risk factors [113,114]. A literature review also provided a similar conclusion: about 75% of patients achieved a good response at 12 months, and 64% achieved remission at 24 months of follow-up [115]. Table 4 highlights the key treatment strategies.

**Table 4:** Treatment strategies for early-onset schizophrenia APS: attenuated psychosis syndrome, EOS: early-onset schizophrenia, LAI: long-acting injectable

Author and year	Mitigation strategies	Key recommendations
Taipale et al. [86]	Addressing clozapine hesitancy	Clozapine showed superior efficacy in treating refractory EOS and the long-term use substantially decreased the mortality
Bosnjak Kuharic et al. [94]	Reducing the duration of untreated psychosis	There are no recommended treatments for APS or prodromal stage
Hickling et al. [109]	Non-adherence	Medication non-adherence was a key predictive factor for relapse
Schoeler et al. [111]	Treatment of cooccurring cannabis use	Cannabis use has been highly associated with medication non-adherence and the risk of relapse
Abdel-Baki et al. [113]	Long-term injectables	LAI antipsychotics significantly reduce the risk of relapse and hospitalizations in ones with poor prognostic risk factors

Challenges within the mental health systems

The evidence suggests the process of diagnosis involves clinical assessment. Neuroimaging is not the standard of care, with many studies recommending against the routine use of magnetic resonance imaging or computed tomography [116,117]. Neuroimaging is only indicated when psychosis due to an organic cause is suspected. [118]. There are issues with managed mental health Medicaid and general medical insurance providers regarding delays in approval for MRI [119]. The diagnostic uncertainty often contributes to a delay in open discussion with patients and their families. The therapeutic alliance between the patient and the family may be affected due to higher degrees of treatment resistance. Assent and consent for treatment are also issues since disagreement with parents with shared custody could delay treatment. Multiple readmissions, a higher risk of completed suicides, and extended durations of inpatient stays could further affect clinical care due to the prevailing purview of managed care organizations to transition into community-level interventions [120]. These transitions are difficult due to the serious shortage [121] of community-based mental health services [122,123] in rural areas [124] and a lack of EIS services. Lastly, healthcare disparity is graver in rural areas, where copayments for expensive antipsychotic medication further affect nonadherence [125]. The practice of denial of medication coverage after patients are discharged from inpatient facilities is not uncommon [126]. The recognized need for more individualized care for EOS is far from reality in large parts of the community. The challenges have been summarized in Table 5.

**Table 5:** Challenges within the mental health systems

Author and year	Key findings
Goulet et al. [116], Kular et al. [117], Forbes et al. [118], Daye et al. [119]	Diagnosis is clinical. Neuroimaging is not the standard of care and is not routinely indicated unless ruling out an organic cause.
Pauselli et al. [120]	Community transition is difficult for families.
Roberts et al. [121], Fontanella et al. [122], Lambert and Agger [123], Summers-Gabr [124]	Community shortages of mental health services in rural USA.
Hensley et al. [125], Neighmond et al. [126]	Healthcare disparity in rural areas includes high copayments and denial of medication coverage upon discharge from inpatient.

## Conclusions

EOS is a serious mental health condition in adolescence. EOS is known for its heterogeneous symptomatology, high DUP, delayed diagnosis, and treatment. Clozapine hesitancy among clinicians has many serious clinical consequences and overall poor outcomes. There is a need to improve predictive approaches with continued education among clinicians and move towards technological advances in precision medicine with the use of neuroimaging and artificial intelligence.
